# Evaluation of the Evolution of Digital Nursing Interventions in an Emergency Unit: An Observational Study

**DOI:** 10.3390/jpm13050712

**Published:** 2023-04-23

**Authors:** Irene Medina Martínez, Juan Carlos Sánchez-García, Jonathan Cortés-Martín, Lourdes Díaz-Rodríguez, María Mercedes Limonchi Pérez, Keyla Vargas Román, Raquel Rodríguez-Blanque

**Affiliations:** 1 School of Health Sciences, University of Granada, 18016 Granada, Spain; irene.medina.mrtnz@gmail.com (I.M.M.); jsangar@ugr.es (J.C.S.-G.);; 2 Research Group CTS-1068, Andalusia Research Plan, Junta de Andalucía, 18014 Granada, Spain; 3 High Resolution Hospital of Loja, 18300 Loja, Spain; 4 University Hospital San Cecilio, 18071 Granada, Spain

**Keywords:** information technology, emergency nursing, electronic health records, nursing records, patient safety

## Abstract

This study aimed to examine the influence of new ICTs on the recording of nursing interventions in the Emergency Unit of the High Resolution Hospital (HRH) of Loja (Granada), Spain. A descriptive observational study was conducted to analyze the evolution of the Nursing Interventions (NIC) records in the Emergency Unit of the Loja HRH (Granada) from 2017 to 2021. Results showed that 11,076 NIC registrations were exploited, which increased by 51.2% from 2017 to 2021. The linear correlation between the NIC and the years was analyzed with Spearman’s coefficient, obtaining a low level of correlation (*p* = 0.166), but one that is statistically significant (*p* < 0.001). The introduction of tablet devices in the emergency room of the Loja HRH (Granada) led to a significant increase in the percentage of NIC recorded and collated during the study period without increasing the number of emergencies attended. However, usability barriers of ICTs were detected, highlighting the need to guide and train health professionals in their use and in the culture of patient safety.

## 1. Introduction

The World Health Organisation (WHO) implemented the Global Strategy for Digital Health in 2020 to help countries strengthen their health systems through the application of Information and Communication Technologies (ICT) [1]. The use of ICT in clinical practice (the main area in nursing) facilitates access to and transfer of information, with patient information, medication delivery, and patient transfer being the most common reasons for their use [2].

Moreover, the use of ICT in clinical practice also allows for remote consultations and telemedicine, which can be especially helpful for patients in rural or remote areas who may not have easy access to healthcare facilities or specialists. With telemedicine, patients can receive medical advice and even have virtual appointments with healthcare professionals without having to travel long distances or leave their homes. This not only saves time and money for patients but also helps to reduce the burden on healthcare facilities and staff [3].

Furthermore, the use of ICT in medication management can help reduce medication errors and improve medication adherence. Electronic prescribing systems can alert healthcare professionals to potential drug interactions, allergies, or dosing errors, while medication reminder apps can help patients remember to take their medications on time [4].

Overall, the implementation of ICT in clinical practice has the potential to improve the quality of care, increase efficiency, and reduce costs in healthcare delivery. However, it is important to ensure that healthcare professionals receive adequate training and support to effectively use these technologies and that patient privacy and confidentiality are maintained in the process [5].

The use of ICT has brought about several positive changes in healthcare delivery. With the help of ICT, healthcare providers can now access and share high-quality information with ease. This has resulted in a significant reduction in the time spent on record-keeping and administrative tasks, freeing up more time for nurses to focus on patient care. Moreover, the implementation of care and evaluation plans has been made more efficient and effective through the use of ICT. This has led to improved nurse outlook and increased patient satisfaction. Overall, the scientific literature has recognized the potential of ICT to enhance healthcare delivery and improve patient outcomes [6,7,8].

The most studied positive effect of ICTs is that of patient safety, as significant results were detected in terms of detection and recording of Adverse Drug Reactions (ADRs) and Adverse Events (AEs), recording of Emergency Department use, and lower rates of infections and falls, among others [9]. Another study found that, with the use of ICT, physiological values were easier to read and interpret, electronic records were completed in less time, and the number of errors in drug administration, blood collection, and specimen transport were reduced [10].

The use of ICT has brought about several positive changes in healthcare delivery. With the help of ICT, healthcare providers can now access and share high-quality information with ease. This has resulted in a significant reduction in the time spent on record-keeping and administrative tasks, freeing up more time for nurses to focus on patient care. Moreover, the implementation of care and evaluation plans has been made more efficient and effective through the use of ICT. This has led to improved nurse outlook and increased patient satisfaction [11,12]. Overall, the scientific literature has recognized the potential of ICT to enhance healthcare delivery and improve patient outcomes.

The integration of Information and Communication Technologies (ICT) into nursing practice and research has become increasingly important in recent years. ICT refers to the use of electronic communication, information management, and technology in the provision of healthcare services.

The use of ICT for healthcare is becoming an increasingly useful tool for healthcare delivery [13]. ICT can be applied in four main areas of nursing: management, teaching, research, and clinical practice. In clinical practice, ICT can improve access to and transfer of quality information, reduce the time spent on records, facilitate the implementation of care and evaluation plans, increase the satisfaction of both nurses and patients, and ensure patient safety, particularly in hospital emergency units [1,2,14]. Due to this, many countries are now including ICT in their nursing curricula; however, they pose a challenge for practicing nurses. Healthcare organizations should thus facilitate continuous training, providing sufficient resources, equipment, and space [9,10,15].

To deepen the theoretical basis of the study it is necessary to highlight the Technology Acceptance Model (TAM). This model is an information systems theory that models how users come to accept and use a technology, providing a theoretical basis for understanding and evaluating user acceptance of new technologies, enabling better systems to be developed and implemented (Venkatesh et al. [16]).

In 2017, in the Loja High Resolution Hospital (HRH) of Granada, a register of interventions was introduced in a computer program called ARIADNA, for the purpose of collating quick interventions to patients, making their registration easier. In 2021, tablet devices were introduced in the Emergency room, enabling nurses to check these interventions in the presence of the patient at the time of their performance.

In 2021, 8163 more emergencies were attended than in 2020, 99.19% of which were resolved without requiring hospital admission [8]. Consequently, it is of great interest to explore whether Information and Communication Technologies (ICTs) facilitated the recording of nursing interventions and decreased the main challenges in the emergency unit: overwhelming workload, limited attention time, difficulty in documenting actions taken, and the undervaluation of nursing roles [9,10]. This study hypothesizes that the introduction of new ICTs will enhance the recording of nursing interventions in the Emergency Unit of the Loja HRH (Granada).

The research question of this article can be stated as: how do new Information and Communication Technologies (ICT) influence the recording of nursing interventions?

The main objective of this study is to determine the influence of new Information and Communication Technologies (ICT) on the recording of Nursing Interventions in the Emergency Department of the High Resolution Hospital (HRH) in Loja (Granada), Spain.

## 2. Materials and Methods

A descriptive observational study was conducted to analyze the evolution of Nursing Interventions records in the Emergency Room of the Loja HRH (Granada), from 20 October 2017 to 31 December 2021. Characteristics such as type of intervention, most prevalent schedule, percentage of interventions unmatched, the relationship to the implementation of new ICT in the unit, and the number of Adverse Events were studied.

An analysis was conducted on all interventions performed on patients who visited the emergency unit during this period, regardless of gender and age. Additionally, data regarding the Adverse Events (AEs) reported to the Patient Safety Observatory in the same period at the Emergency unit of the Loja HRH (Granada) were utilized.

As indicated above, the present study was conducted during the period from October 2017 to December 2021. During this period, the Emergency Department of the Loja HRH (Granada) attended to a total of 185,039 patients. Of the total number of patients attended, 11,076 patients were included in the study, applying the methodology presented here.

One of the aspects to be taken into account in the evaluation of these data is the increase in the number of patients included in the study according to the chronological evolution of the study. This shows the integration and maturity of the project among the service’s professionals. In addition, it should also be noted that during the years of maximum tension and critical state of the COVID-19 pandemic, the study continued to show the highest registration figures of the entire study. A clearer and more detailed presentation of the sample size is presented in the Section 3.

In order to conduct this study, a bibliographic review of the scientific literature was initially conducted to analyze the current state of ICTs and nursing intervention registries, as well as their influence on patient safety. Various articles were obtained from health sciences databases Scopus, Medline, and PubMed, using the descriptors DeCS (Descriptors in Health Sciences) “Information Technology”, “Emergency nursing”, “Electronic Health Records”, “Nursing Records” and “Patient Safety”, with the help of the Boolean operators “AND” and “OR”. After a comprehensive inspection of the titles and abstracts of the articles, those that did not directly relate ICT to nursing intervention registries in the emergency area were excluded. Nevertheless, other articles from other healthcare settings such as critical care and hospitalization were still taken into account.

The inclusion criteria for the different articles were time-limited to the last 5 years (2017–2022) and language (Spanish or English). Ten articles were ultimately referenced in the bibliography and used to establish the theoretical framework of this work, among them, the study on the Technology Acceptance Model (TAM) by Venkatesh et al. [16] stands out. Additionally, an anonymous database was created in conjunction with the list of Adverse Events (AEs) reported in the Patient Safety Observatory at the Hospital Real de Granada, provided by the Nursing Supervisor of the Emergency Unit. Finally, a database was compiled, along with statistical analysis.

After obtaining authorization from the Center and the Granada Provincial CEI, the anonymized data was utilized for analysis, ensuring confidentiality at all times. No Informed Consent was needed as the study did not pose any risk or benefit to any person, since it was done using a database and there was no direct contact with the patients.

The data were extracted from the Patient Safety Observatory of the Hospital Real de Granada by prior request and presentation of the study developed here. The principal investigator of the study, accompanied by the person responsible for the Nursing Supervision of the Emergency Unit, was in charge of this process. The platform used as a database is digitally based at https://www.juntadeandalucia.es/organismos/saludyconsumo/areas/calidad-investigacion-conocimiento/calidad-sistema-sanitario/paginas/seguridad-pacientes.html (accessed on 2 February 2023). All the confidentiality data of this process had the ethical endorsement and responsibility of the aforementioned persons responsible for this study.

The outcomes studied were: Gender (Male/Female), Intervention (NIC) with the following codes: 2313 (Administration of medication: Intramuscular (IM)), 2314 (Administration of medication: Intravenous (IV)), 2304 (Medication administration: Oral), 2317 (Medication administration: Subcutaneous (SC)), 2315 (Medication administration: Rectal), 3660 (Wound care), 3620 (Suture), 0910_1 (Immobilization: Prim splint), 0910_2 (Immobilization: Anterior/posterior lower limb splint (MMII)), 0910_3 (Immobilization: Anterior/posterior upper limb splint (MMSS)), 0910_4 (Immobilization: Limb bandaging), 0910_5 (Immobilization: Other immobilizations), 632025 (Electrocardiogram (ECG)), 0580 (Bladder catheterization), 1080 (Gastrointestinal probing), 4235 (Phlebotomy: Channeled route), 4238 (Phlebotomy: Blood sample), 6680 (Monitoring of vital signs), 7820 (Sample handling), Year (2017 to 2021), Hourly interval (three intervals, corresponding to the shifts worked by the nursing staff at the Granada HAR), and Intervention completed (Yes/No).

The variables of gender, interventions, and time interval were used to characterize Loja HRH (Granada) and the most prevalent interventions. Additionally, the variables of year and completed intervention were used to answer the main objective of the study. All variables were converted into numerical values for statistical analysis. Integer numerical values between 0 and 18 were assigned for each intervention of the NIC variable, 0 and 1 for gender and intervention performed, between 0 and 4 for the year variable, between 0 and 2 for time interval and 0–11 for the months of the year.

A database was created from the data collected from the ARIADNA program, using Microsoft Excel. SPSS Statistics 28 was used to conduct all statistical calculations. A descriptive analysis of the variables was carried out, with the use of frequency and percentage. Furthermore, to assess the correlation between the implementation of new ICTs and the rise in the number of interventions registered and collated, a comparison was made between the NICs collated prior to the introduction of tablets and after their use. Spearman’s coefficient was utilized for this purpose, with a 95% confidence interval and a statistical significance of less than 0.05 (*p* < 0.05).

## 3. Results

The main results of this study are presented below. A total of 185,039 patients were seen in the Emergency Department of the Loja HRH (Granada) during the period of time that the study was implemented (Table 1). As indicated in the Section 2, the following table concisely shows the development and breadth of the sample.

Between the years 2017 and 2021, a total of 11,076 nursing interventions (NIC) were recorded. The NIC registry was first implemented in 2017, and the amount of interventions steadily increased from there, with 725 (6.5% of the total) in 2017 and 6389 (57.7% of the total) in 2021. These records do not include nursing interventions conducted when patients were admitted to the observation area, or those related to collecting nasopharyngeal exudate samples for the detection of SARS-CoV-2 virus (antigen test or PCR). Table 2 shows the frequency and percentage of nursing interventions between 2017 and 2021.

Analysis of the NIC records revealed that 48.9% (n = 5421) of the interventions during the study period were performed on males and 51.1% (n = 5655) were performed on females. The afternoon shift (15:00–21:59 h) had the highest number of NICs at 46.3% (n = 5133), followed by the morning shift (8:00–14:59 h) at 40.3% (n = 4459). The night shift (22:00–7:59 h) had the lowest number of NICs, with a percentage of 13.4% (n = 1484). Table 3 shows the frequency and percentage of interventions according to the morning, afternoon, or night shift.

From the records of NIC in the different time intervals, the number and percentage of interventions that were recorded by the nurses were analyzed (Table 4). The time interval with the highest number of NICs was the morning, at 68.2% (n = 3043), followed by the afternoon at 66.5% (n = 3411). The shift with the least amount of NICs was the night shift, at 59.4% (n = 882).

Supplementary Table S1 displays the total number of registered Nursing Interventions (NICs) implemented in the ARIADNA Program’s Intervention Registry. 19 NICs are organized from the highest to the lowest number of registrations. The prevalence of NICs was studied from 2017 to 2021, with the three most performed being “2313 Medication Administration: Intramuscular (IM)”, “0910_4 Immobilization: Extremity Dressing”, and “3660 Wound Care”, accounting for 61.7%, 9%, and 7.1%, respectively.

In the ARIADNA program’s Interventions register, the “Completed” option should be checked by the nurse performing the intervention. This option was enabled when the tablets were introduced in 2021, allowing the procedure to be compared in the same place and at the same time. Table 5 shows the evolution of matched and unmatched NIC in frequency and percentage.

Supplementary Table S2 shows that the correlation between the Nursing Interventions Care (NIC) variable and the year variable was analyzed with Spearman’s coefficient, obtaining a low level of correlation (*p* = 0.166), being closer to 0 than to 1, and positive. This suggests that, over the years and with the implementation of tablets in the last year, the percentage of NICs collated has increased, although this correlation was not linear. The result was statistically significant (*p* < 0.001), meaning that the correlation established is very likely true and, therefore, the null hypothesis (H₀), formulated as “there are no differences between the implementation of new ICT and the recording of Nursing Interventions in the Emergency Unit of the HRH of Granada”, is rejected. Table 6 shows that, over the past five years, more than 97% of cases in the Loja HRH (Granada) did not require admission. Of those non-admitted EUs, the percentage of NIC recorded in ARIADNA has been increasing, reaching 17.37% in 2021—coinciding with the implementation of tablets in the Emergency Unit.

Finally, Supplementary Table S3 shows the adverse events (AE) recorded by the Patient Safety Observatory during the study period. The table displays the adverse events along with the dates on which they occurred. A total of 22 results are shown in Supplementary Table S3. The relationship between recorded NICs and the number of AE’s was not analyzed because, in the last four years, AE’s occurring in the Emergency Unit have not been systematically reported.

## 4. Discussion

This study aimed to determine the influence of new Information and Communication Technologies (ICT) on the recording of Nursing Interventions in the Emergency Department of the High Resolution Hospital (HAC) of Granada.

The nursing process is an essential part of health services and it is defined as a set of actions based on scientific knowledge, technical skills, social skills, and attitudes for providing nursing care. Among these competences, ICT (Information and Communication Technologies) and technological resources have been progressively implemented in healthcare over the years. Hospitals have invested heavily to implement and improve new ICT, such as the ARIADNA software in the Loja HRH (Granada). The software was improved in 2017 by introducing a register with different NICs and in 2021, tablets were introduced to facilitate registration during interventions. This study showed that the percentage of NICs registered and collated has increased over the years, as seen in Table 1 and Table 4, and Supplementary Table S2, and that there was no increase in emergency interventions, as seen in Table 5.

The implementation of new ICTs can lead to reduced hospital costs due to increased efficiency in providing care, improved quality, and safety. This is because the number of typing errors and adverse outcomes is reduced and information is exchanged more quickly [12]. This study aimed to evaluate the impact of new ICTs on patient safety, based on the adverse events reported to the Patient Safety Observatory (Supplementary Table S3). It is true that the number of reported adverse events has decreased, however this is likely due to the fact that they have not been reported systematically in the last four years. Thus, it is not possible to establish a causal relationship between the implementation of ICT and adverse events in the unit.

The Regional Study of Adverse Events Derived from Care (ERIDA) conducted in 2017 showed that the most frequent adverse events were related to medication and, in second place, to care, with 60% of these being considered avoidable [7]. These results were similar to those of the 2010 national study of Adverse Events in the Emergency unit (EVADUR) [8]. This study analyzed the most common Nursing Interventions in the Emergency Unit of the Loja (Granada) Hospital, with the administration of intramuscular medication (61.7%) at the top, which can be seen in Supplementary Table S1. The use of tablets over the last year has made it easier to check drugs, dosage, route of administration, and the patient’s drug allergies and intolerances prior to administration, and to document this intervention in front of the patient, which may also lead to improved patient safety.

In addition to improved quality of care through increased patient safety, ICT brings other benefits to clinical practice. According to the scientific literature, ICTs can increase efficiency, speed, and access to information [2,6,13,14], which are important characteristics for an Emergency Unit due to the high volume of patients and care load. However, the results show that 13.4% of attendances occur during the night shift, which has the lowest number of emergencies attended and the lowest percentage of NICs recorded (59.4%) (Table 3). Therefore, records of NICs performed by nurses are not only influenced by the amount of care, but also by lack of motivation, training, or orientation, which are barriers that should be addressed by healthcare organizations, as described in other studies [11,15].

The main limitations of this study, and why the results could be biased, are as follows:

- This study was performed at the Loja HRH (Granada), which may not be representative of other hospitals in Andalusia due to the different ARIADNA software and work methodology used.
- Difficulty in obtaining patient safety data due to the lack of notification of Adverse Events to the Patient Safety Observatory.
- Technology being used as an assistive tool to improve people’s conditions can influence multiple aspects of assistance and care, but lack of management and operational problems can cause stress and anxiety in nurses.
- The use of technology for training and education of nursing professionals can help them to gain skills, adapt to the work environment, and foster interest in developing projects that utilize the technology. However, the nurse’s actions will determine if the technology is used in a positive or negative way.

## 5. Conclusions

In conclusion, the use of ICT has been shown to improve the quality and efficiency of services and care, increase patient safety, facilitate the management of communication, improve documentation, and streamline the management and tracking of information. However, it was also identified that health professionals need more guidance and training in order to use ICT effectively and to its fullest potential. Furthermore, this would provide a more accurate representation of the work done by nurses in Emergency Units, and would add value to their profession.

The data obtained in this study show that the implementation of ICT in nursing practice improves the quality of the records of their interventions and optimises their clinical practice in emergency situations.

## Figures and Tables

**Table 1:** Sample size and registers.

Year	Total Attendance	Registry	Cumulative Percentage (%)
2017	38,204	725	1.90
2018	39,537	363	0.92
2019	41,291	1591	3.85
2020	28,922	2008	6.94
2021	37,085	6389	17.23
Total	185,039	11,075	30.84

**Table 2:** Frequency and percentage of nursing interventions in the period from 2017–2021.

Year	Frequency (n)	Percentage (%)	Cumulative Percentage (%)
2017	725	6.5	6.5
2018	363	3.3	9.8
2019	1591	14.4	24.2
2020	2008	18.1	42.3
2021	6389	57.7	100
Total	11,076	100	100

**Table 3:** Frequency and percentage of interventions according to the nursing shift (morning, afternoon, or evening).

Hourly Interval	Frequency (*n*)	Percentage (%)	Cumulative Percentage (%)
Morning shift 8–14:59 h	4459	40.3	40.3
Afternoon shift 15–21:59 h	5133	46.3	86.6
Night shift 22–7:59 h	1484	13.4	100
Total	11,076	100	100

**Table 4:** Frequency and percentage of NIC collated in the different nursing shifts (morning, afternoon, or evening).

Hourly Interval	NIC Collated (*n*)		Total (*n*)	Percentage NIC Collated (%)	
	No	Yes		No	Yes
8–14:59	1416	3043	4459	31.8	68.2
15–21:59	1721	3411	5133	33.5	66.5
22–7:59	602	882	1484	40.6	59.4

**Table 5:** Evolution of collated/uncollated NICs in frequency and percentage, in the time period between 2017–2021.

Year	NIC Not Matched		Collated NIC		Total (*n*)
	Frequency (*n*)	Percentage (%)	Frequency (*n*)	Percentage (%)	
2017	348	48.0	377	52.0	725
2018	210	57.9	153	42.1	363
2019	464	29.2	1124	70.8	1588
2020	1038	51.7	968	48.3	2006
2021	1668	26.2	4704	73.8	6372
Total	3728	33.7	7326	66.3	11,054

**Table 6:** Emergencies attended, frequency and percentage of Emergencies not admitted, and percentage of Emergencies with NIC recorded from 2017–2021.

Year	Emergencies Attended (*n*)	Percentage of Not Admitted Emergencies (%)	NICs Registered with ARIADNA (*n*)	Non-Admitted Emergencies (*n*)	Percentage of Emergencies with NIC Registered in ARIADNA (%)
2017	38,204	98	725	37,455	1.9
2018	39,537	97.9	363	38,715	0.9
2019	41,291	98.3	1591	40,589	3.9
2020	28,922	99.2	2008	28,699	7
2021	37,085	99.2	6389	36,785	17.4

## Data Availability

Data are available on request from the corresponding author.

## Supplementary Materials

The following supporting information can be downloaded at: https://www.mdpi.com/article/10.3390/jpm13050712/s1. Table S1 shows the frequency and percentage of NICs implemented in the registry of interventions of the ARIADNA program. Table S2 presents the correlation between matched NICs and year, according to Spearman’s coefficient. Table S3 displays adverse events reported to the Patient Safety Observatory during the study period.

## References

[1] Brenner S.K., Kaushal R., Grinspan Z., Joyce C., Kim I., Allard R.J., Delgado D., Abramson E.L. (2016). Effects of Health Information Technology on Patient Outcomes: A Systematic Review. J. Am. Med. Inform. Assoc..

[2] Lee T.-Y., Sun G.-T., Kou L.-T., Yeh M.-L. (2017). The Use of Information Technology to Enhance Patient Safety and Nursing Efficiency. Technol. Health Care.

[3] Haleem A., Javaid M., Singh R.P., Suman R. (2021). Telemedicine for Healthcare: Capabilities, Features, Barriers, and Applications. Sens. Int..

[4] Agrawal A. (2009). Medication Errors: Prevention Using Information Technology Systems. Br. J. Clin. Pharmacol..

[5] Lluch M. (2011). Healthcare Professionals’ Organisational Barriers to Health Information Technologies—A Literature Review. Int. J. Med. Inform..

[6] Jha A.K., Doolan D., Grandt D., Scott T., Bates D.W. (2008). The Use of Health Information Technology in Seven Nations. Int. J. Med. Inform..

[7] Hsiao C.-J., Hing E., Socey T.C., Cai B. (2011). Electronic Health Record Systems and Intent to Apply for Meaningful Use Incentives among Office-Based Physician Practices: United States, 2001–2011.

[8] van Houwelingen C.T.M., Moerman A.H., Ettema R.G.A., Kort H.S.M., ten Cate O. (2016). Competencies Required for Nursing Telehealth Activities: A Delphi-Study. Nurse Educ. Today.

[9] Borycki E.M., Cummings E., Kushniruk A.W., Saranto K. (2017). Integrating Health Information Technology Safety into Nursing Informatics Competencies. Stud. Health Technol. Inform..

[10] Morales Arandojo M.I., Martín Conty J.L. (2017). Las TIC En La Enfermería Docente. ENE Rev. Enfermería.

[11] Huter K., Krick T., Domhoff D., Seibert K., Wolf-Ostermann K., Rothgang H. (2020). Effectiveness of Digital Technologies to Support Nursing Care: Results of a Scoping Review. J. Multidiscip. Healthc..

[12] Alotaibi Y.K., Federico F. (2017). The Impact of Health Information Technology on Patient Safety. Saudi Med. J..

[13] Rouleau G., Gagnon M.-P., Côté J., Payne-Gagnon J., Hudson E., Dubois C.-A. (2017). Impact of Information and Communication Technologies on Nursing Care: Results of an Overview of Systematic Reviews. J. Med. Internet Res..

[14] Askari-Majdabadi H., Valinejadi A., Mohammadpour A., Bouraghi H., Abbasy Z., Alaei S. (2019). Use of Health Information Technology in Patients Care Management: A Mixed Methods Study in Iran. Acta Inform. Med..

[15] Konttila J., Siira H., Kyngäs H., Lahtinen M., Elo S., Kääriäinen M., Kaakinen P., Oikarinen A., Yamakawa M., Fukui S. (2019). Healthcare Professionals’ Competence in Digitalisation: A Systematic Review. J. Clin. Nurs..

[16] Venkatesh V., Morris M.G., Davis G.B., Davis F.D. (2003). User Acceptance of Information Technology: Toward a Unified View. MIS Q..

