# Supplementary material for: Evaluation of the Evolution of Digital Nursing Interventions in an Emergency Unit: An Observational Study
Source: J Pers Med. 2023 Apr 23;13(5):712. doi: 10.3390/jpm13050712 (PMC10221346; doi:10.3390/jpm13050712)
Supplement: Supplementary file 1 [file jpm-13-00712-s001.zip › jpm-2321400-supplementary.pdf]

Supplementary Table S1: Frequency and percentage of NICs implemented in the registry of interventions of the ARIADNA program.

**Nursing Intervention (code and NIC)**	**Frequency *(n)***	**Percentage *(%)***
2313 Medication Administration: Intramuscular (IM)	6830	61.7
0910_4 Immobilization: Limb bandaging	993	9.0
3660 Wound care	787	7.1
3620 Suture	631	5.7
2304 Medication administration: Oral	419	3.8
0910_2 Immobilization: Anterior/Posterior splint of MMII	287	2.6
0910_3 Immobilization: Anterior/Posterior Splint of MMSS	281	2.5
0910_5 Fixed assets: Other fixed assets	210	1.9
0910_1 Immobilization: Prim Splint	169	1.5
2317 Medication Administration: Subcutaneous (SC)	105	0.9
2314 Medication Administration: Intravenous (IV)	93	0.8
2315 Medication administration: Rectal	69	0,6
0580 Bladder catheterization	61	0.6
632025 Performance of electrocardiogram (ECG)	48	0.4
4238 PHLEBOTOMY: BLOOD SAMPLING	37	0.3
7820 SAMPLE HANDLING	23	0,2
4235 PHLEBOTOMY: CANNULATED ROUTE	14	0.1
6680 VITAL SIGNS MONITORING	12	0,1
1080 GASTROINTESTINAL CATHETERIZATION	7	0.1
**TOTAL**	11076	100

Supplementary Table S2: Correlation between the NICs matched and the year with Spearman's coefficient.

			**Year**	**Completed**
Spearman's Rho	Year	Correlation coefficient	1000	**0.166****
		p	.	**< 0.001**
		n	11076	11054
	Collated NIC	Correlation coefficient	0,166**	1,000
		p	< 0.001	.
		n	11054	11054
** The correlation is significant at the 0.01 level (bilateral).				

Supplementary Table S3. Adverse events reported to the Patient Safety Observatory during the study period.

Date	Adverse Event
16/01/2017	Serum systems
21/01/2017	Serum systems
17/05/2017	Charging needles
25/07/2017	Peripheral catheter
7/08/2017	Adverse reaction to ciprofloxacin
7/08/2017	Peripheral catheter
15/08/2017	Change of location of vacutainer bells
7/10/2017	Digoxin prescription
25/10/2017	Insulin administration
23/11/2017	Allergy to diclofenac
29/11/2017	Nebulization error (medical air)
27/12/2017	Electrocardiogram clamp injury
27/12/2017	Duplicity in the regimen of Tamsulosin
17/02/2018	Diclofenac misplaced in Omnicell cabinet
23/02/2018	Error in transfer from the DCCU to the hospital (administration of drugs)
1/04/2018	Wrong location of Nitroglycerin IV in Critical Care Box
22/04/2018	Metamizole allergy
2/06/2019	Provision of regular unlabeled medication
18/11/2019	Phlebitis due to medication extravasation
18/12/2020	Accidental application of tissue adhesive in the eye
23/02/2021	Wrong administration of rabies vaccine in place of tetanus vaccine
17/11/2021	Transport of samples in critical care ambulance
